# Ochratoxin A Dietary Exposure of Ten Population Groups in the Czech Republic: Comparison with Data over the World

**DOI:** 10.3390/toxins7093608

**Published:** 2015-09-10

**Authors:** Vladimir Ostry, Frantisek Malir, Marcela Dofkova, Jarmila Skarkova, Annie Pfohl-Leszkowicz, Jiri Ruprich

**Affiliations:** 1 National Reference Center for Microfungi and Mycotoxins in Food Chains, Center of Health, Nutrition and Food in Brno, National Institute of Public Health in Prague, 61242 Brno, Czech Republic; E-Mails: dofkova@chpr.szu.cz (M.D.); j.skark@seznam.cz (J.S.); jruprich@chpr.szu.cz (J.R.); 2 Department of Biology, Faculty of Science, University of Hradec Kralove, 50003 Hradec Kralove, Czech Republic; E-Mail: frantisek.malir@uhk.cz; 3 Department Bioprocess & Microbial Systems, Laboratory Chemical Engineering, INP/ENSA Toulouse, University of Toulouse, UMR 5503 CNRS/INPT/UPS, 31320 Auzeville-Tolosane, France; E-Mail: leszkowicz@ensat.fr

**Keywords:** ochratoxin A, food, exposure sources, dietary exposure, Czech population

## Abstract

Ochratoxin A is a nephrotoxic and renal carcinogenic mycotoxin and is a common contaminant of various food commodities. Eighty six kinds of foodstuffs (1032 food samples) were collected in 2011–2013. High-performance liquid chromatography with fluorescence detection was used for ochratoxin A determination. Limit of quantification of the method varied between 0.01–0.2 μg/kg depending on the food matrices. The most exposed population is children aged 4–6 years old. Globally for this group, the maximum ochratoxin A dietary exposure for “average consumer” was estimated at 3.3 ng/kg bw/day (lower bound, considering the analytical values below the limit of quantification as 0) and 3.9 ng/kg bw/day (middle bound, considering the analytical values below the limit of quantification as 1/2 limit of quantification). Important sources of exposure for this latter group include grain-based products, confectionery, meat products and fruit juice. The dietary intake for “high consumers” in the group 4–6 years old was estimated from grains and grain-based products at 19.8 ng/kg bw/day (middle bound), from tea at 12.0 ng/kg bw/day (middle bound) and from confectionery at 6.5 ng/kg bw/day (middle bound). For men aged 18–59 years old beer was the main contributor with an intake of 2.60 ng/kg bw/day (“high consumers”, middle bound). Tea and grain-based products were identified to be the main contributors for dietary exposure in women aged 18–59 years old. Coffee and wine were identified as a higher contributor of the OTA intake in the population group of women aged 18–59 years old compared to the other population groups.

## 1. Introduction

Ochratoxin A (OTA) is a nephrotoxic and renal carcinogenic mycotoxin [1,2,3]. OTA is produced by *Aspergillus* and *Penicillium* species [4,5,6,7] and is a common contaminant of various foodstuffs of plant and animal origin including cereals, spices, coffee, cacao, beer, wine, raisins, pulses, meat, meat products, or edible offal [7].

Considering such ubiquity and the mentioned toxic effects, international authorities have proposed tolerable daily or weekly intakes for OTA, which indicate the dose that can be safely consumed daily/weekly over a lifetime without incurring any appreciable adverse health effects. Tolerable Weekly Intake (TWI) of 120 ng/kg bw/week was established for OTA based on the ground of its nephrotoxic properties in pig. TWI was established on the basis of the lowest observed adverse effect level (LOAEL) of 8 μg/kg bw/day for early markers of renal toxicity in pigs (the most sensitive animal species), and applying a composite uncertainty factor of 450 for the uncertainties in the extrapolation of experimental data derived from animals to humans as well as for intra-species variability [8]. Based on carcinogenic properties of OTA, the negligible cancer risk intake has been assessed to be 4 ng OTA/kg bw/day by Health Canada [9] and the value of 5 ng/kg bw/day was already proposed by the French Agency for Food Safety as early as in 1999 [10].

The current paper aims to present the data on the dietary exposure assessment of OTA with respect to 10 sex-age groups in the Czech Republic (CR) as investigated in the research project No. NT12051–3/2011 entitled “Ochratoxin A–health risk assessment for selected population groups in the Czech Republic” [11]. The rationale behind the project solution is recent knowledge on OTA carcinogenicity (OTA adducts with DNA), cumulative effects of OTA in organisms, information on the large number of OTA exposure sources, recent results of urinary OTA excretion in men and women in the Czech Republic and incomplete data on OTA dietary exposure with respect to population in CR [3,11].

## 2. Results

### 2.1. Presence of OTA in Food

All the data are presented in alphabetical order by food items (Table 1, Table 2 and Table 3). Table 1 summarizes the analytical data of the samples presenting every time OTA.

**Table 1:** Food samples with OTA.

Food	*n*	n+%	Arithmetic Mean (μg/kg)	Range Min–Max (μg/kg)	LOQ (μg/kg)
Beer, regular, lager ^a^	12	100	0.064	0.02–0.18	0.01
Liquorice root dried	12	100	15.80	3.80–36.70	0.2
Nutmeg	12	100	8.70	0.30–60.70	0.2
Paprika powder, fiery ^b^	12	100	19.00	5.50–91.80	0.2
Paprika powder, sweet	12	100	16.00	1.10–38.40	0.2

Notes: LOQ: limit of quantification; *n*: number of samples; n+%: percent of positive samples >LOQ; ^a^ beer, regular, lager contains 5% alcohol; ^b^ with low concentration of capsaicin than in chili pepper.

The analytical results (mean and range) concerning the occurrence of OTA in at least one sample of foodstuffs of plant and animal origin are presented in Table 2.

**Table 2:** Food samples with OTA (positive at least in one sample).

Food	*n*	n+%	Arithmetic Mean (μg/kg)			Range Min–Max (μg/kg)	LOQ (μg/kg)
			LB	MB	UB		
Beans	12	8	8.90	9.00	9.10	0.0–107	0.2
Beer, regular ^a^	12	83	0.065	0.066	0.067	0.0–0.26	0.01
Bird offal	12	8	0.02	0.12	0.21	0.0–0.28	0.2
Biscuits, sweet, plain	12	58	0.53	0.57	0.61	0.0–1.69	0.2
Bitter chocolate	12	42	0.23	0.29	0.35	0.0–1.01	0.2
Black pepper	12	92	0.83	0.83	0.84	0.0–2.82	0.2
Breakfast cereals	12	33	0.17	0.23	0.30	0.0–0.67	0.2
Biscuits chocolate	12	75	0.20	0.24	0.27	0.0–0.56	0.2
Caraway	12	17	0.11	0.19	0.28	0.0–0.71	0.2
Cereal bars	12	25	0.12	0.19	0.27	0.0–0.61	0.2
Cereal porridge	12	8	0.04	0.13	0.22	0.0–0.44	0.2
Chicken fresh meat	12	8	0.03	0.12	0.22	0.0–0.38	0.2
Chili pepper, dried	12	92	6.69	6.70	6.70	0.0–32.7	0.2
Chocolate coated confectionery	12	50	0.24	0.29	0.34	0.0–1.16	0.2
Cocoa beverage-preparation, powder	12	8	0.04	0.13	0.22	0.0–0.45	0.2
Cocoa powder	12	50	0.89	0.94	0.99	0.0–4.10	0.2
Coffee ground, roasted	12	58	0.37	0.41	0.45	0.0–1.04	0.2
Coriander seed	12	33	0.39	0.46	0.53	0.0–1.96	0.2
Crackers	12	8	0.04	0.13	0.22	0.0–0.45	0.2
Dried figs	12	8	0.08	0.17	0.27	0.0–0.99	0.2
Dried wine fruits (raisins)	12	42	0.40	0.46	0.52	0.0–2.17	0.2
Filled chocolate	12	17	0.04	0.13	0.21	0.0–0.33	0.2
Fine bakery wares	12	42	0.08	0.14	0.20	0.0–0.21	0.2
French bread	12	8	0.02	0.11	0.20	0.0–0.20	0.2
Fruit juices	12	58	0.023	0.025	0.027	0.0–0.059	0.01
Ginger root dried	12	58	2.00	2.04	2.09	0.0–12.7	0.2
Gingerbread	12	58	0.41	0.45	0.49	0.0–1.44	0.2
Green tea leaves and stalks (dry)	12	83	1.90	1.96	2.02	0.0–8.50	0.2
Herbs and vegetables for infusions—fruit tea	12	33	8.93	8.98	9.07	0.0–104	0.2
Herbs and vegetables for infusions—herbal tea	12	8	0.14	0.23	0.32	0.0–1.62	0.2
Instant coffee	12	92	1.03	1.04	1.05	0.0–4.91	0.2
Milk chocolate	12	33	0.11	0.17	0.24	0.0–0.40	0.2
Mixed herbs and spices 1	12	83	1.62	1.64	1.65	0.0–9.40	0.2
Mixed herbs and spices 2	12	8	0.03	0.13	0.22	0.0–0.40	0.2
Muesli	12	17	0.14	0.23	0.31	0.0–1.44	0.2
Multigrain bread and rolls	12	17	0.05	0.14	0.22	0.0–0.34	0.2
Mustard	12	25	0.05	0.13	0.21	0.0–0.37	0.2
Oat rolled grains	12	8	0.02	0.11	0.20	0.0–0.24	0.2
Pork, fresh	12	8	0.02	0.11	0.20	0.0–0.20	0.2
Pork kidney	12	8	0.04	0.13	0.22	0.0–0.46	0.2
Pasta and similar products	12	8	0.05	0.14	0.23	0.0–0.55	0.2
Rice grains	12	8	0.31	0.41	0.50	0.0–3.76	0.2
Rye bread, refined flour	12	25	0.08	0.16	0.23	0.0–0.61	0.2
Rye-wheat bread, refined flour	12	33	0.12	0.19	0.27	0.0–0.81	0.2
Sponge biscuits, plain	12	58	0.36	0.41	0.46	0.0–2.14	0.2
Sweet confectionery	12	83	0.65	0.67	0.68	0.0–1.78	0.2
Tea leaves and stalks, fermented	12	33	32.90	33.00	33.10	0.0–250	0.2
Wine, red	12	25	0.065	0.069	0.073	0.0–0.695	0.01
Wine, white	12	42	0.014	0.017	0.020	0.0–0.036	0.01

Notes: *n*: number of samples; n+%: percent of positive samples >LOQ; LB—lower bound (analytical data <LOQ = 0); MB—middle bound (analytical data <LOQ = LOQ/2); UB—upper bound (analytical data <LOQ = LOQ); ^a^ beer, regular contains alcohol below 4%.

Food samples of plant and animal origin without OTA occurrence (all food samples were below LOQ) are presented in Table 3.

**Table 3:** Food samples of plant and animal origin without OTA.

Food	*n*	n+%	Arithmetic Mean (μg/kg)	Range Min–Max (μg/kg)	LOQ (μg/kg)
Blood-type sausage	12	0	-	-	0.2
Bovine fresh meat	12	0	-	-	0.2
Breadcrumbs	12	0	-	-	0.2
Canned meat	12	0	-	-	0.2
Chestnut	12	0	-	-	0.2
Chicken fresh meat	12	0	-	-	0.2
Frankfurter sausage	12	0	-	-	0.2
Ginger root fresh	12	0	-	-	0.2
Ham, pork 1	12	0	-	-	0.2
Ham, pork 2	12	0	-	-	0.2
Hazelnuts	12	0	-	-	0.2
Head cheese pork	12	0	-	-	0.2
Hen eggs	12	0	-	-	0.2
Hungarian-type salami	12	0	-	-	0.2
Knackwurst-type sausage	12	0	-	-	0.2
Lentil (dry)	12	0	-	-	0.2
Liver based spreadable	12	0	-	-	0.2
Liver-type sausage	12	0	-	-	0.2
Luncheon meat	12	0	-	-	0.2
Multigrain rolls	12	0	-	-	0.2
Pate, pork liver	12	0	-	-	0.2
Peas (dry)	12	0	-	-	0.2
Polish-type cooked sausage 1	12	0	-	-	0.2
Polish-type cooked sausage 2	12	0	-	-	0.2
Pork liver	12	0	-	-	0.2
Smoked cooked sausage	12	0	-	-	0.2
Walnuts	12	0	-	-	0.2
Wheat flour fine	12	0	-	-	0.2
Wheat flour medium-coarse	12	0	-	-	0.2
Wheat flour coarse	12	0	-	-	0.2
Wheat semolina	12	0	-	-	0.2

Notes: *n*: number of samples; n+%: percent of positive samples >LOQ.

Fifty kinds (82%) of food of 61 kinds of food of plant origin in total were found to contain OTA. The main OTA sources included cereals, spices, coffee, tea, wine, and beer. Except for beer lager, dried liquorice root, nutmeg, fiery paprika powder and sweet paprika powder which are always contaminated, the mean occurrence of OTA in the analyzed food samples (positive at least in one sample) was 36%.

As for OTA occurrence in food samples of animal origin, four kinds (17%) of 23 kinds in total (pork fresh meat, swine kidney, chicken fresh meat and bird offal) contained very low amount of OTA.

### 2.2. Dietary Exposure Assessment

Estimation of dietary exposure to OTA for average consumers with respect to each groups (ng/kg bw/day) are presented in Table 4, Table 5 and Table 6. The values assessed has been done using the national wide dietary survey (SISP04).

**Table 4:** Estimation of the average exposure of OTA in children consumers (ng/kg bw/day).

Food group	Population group					
	Children 4–6 years			Children 7–10 years		
	LB	MB	UB	LB	MB	UB
Cocoa	0.020	0.040	0.060	0.013	0.021	0.030
Coffee	0.002	0.002	0.002	0.001	0.001	0.002
Confectionery	0.240	0.270	0.310	0.160	0.200	0.210
Dried fruit	0.008	0.010	0.012	0.002	0.003	0.003
Grains and grain-based products	0.750	1.100	1.420	0.560	0.820	1.100
Juice	0.120	0.130	0.140	0.070	0.080	0.090
Meat and meat products	0.040	0.180	0.340	0.040	0.160	0.300
Spices, seasoning and legumes	0.210	0.210	0.220	0.210	0.210	0.210
Tea	1.910	1.920	1.940	1.130	1.140	1.150
Beer	0	0	0	0	0	0
Wine	0	0	0	0	0	0
Total	3.3	3.9	4.4	2.2	2.6	3.1

Notes: LB—lower bound (analytical data <LOQ = 0); MB—middle bound (analytical data <LOQ = LOQ/2); UB—upper bound (analytical data <LOQ = LOQ).

**Table 5:** Estimation of average exposure of OTA in adolescent consumers (ng/kg bw/day).

Food group	Population Group											
	Boys 11–14 years			Girls 11–14 years			Men 15–17 years			Women 15–17 years		
	LB	MB	UB	LB	MB	UB	LB	MB	UB	LB	MB	UB
Cocoa	0.007	0.012	0.017	0.012	0.018	0.025	0.008	0.011	0.014	0.002	0.004	0.006
Coffee	0	0	0	0.001	0.001	0.002	0.002	0.002	0.003	0.007	0.008	0.0082
Confectionery	0.090	0.100	0.114	0.100	0.110	0.120	0.030	0.040	0.050	0.035	0.040	0.050
Dried fruit	0.002	0.002	0.002	0.001	0.001	0.001	0	0	0	0.004	0.006	0.009
Grains and grain-based products	0.540	0.790	1.050	0.450	0.650	0.900	0.500	0.720	0.100	0.200	0.380	0.520
Juice	0.012	0.013	0.014	0.040	0.040	0.050	0.030	0.030	0.040	0.020	0.030	0.030
Meat and meat products	0.030	0.130	0.240	0.040	0.160	0.300	0.030	0.150	0.270	0.025	0.120	0.210
Spices, seasoning and legumes	0.160	0.160	0.170	0.145	0.150	0.153	0.159	0.160	0.170	0.110	0.110	0.120
Tea	0.890	0.890	0.900	0.840	0.850	0.850	0.620	0.620	0.630	0.560	0.570	0.580
Beer	0	0	0	0	0	0	0.030	0.030	0.031	0.034	0.034	0.0343
Wine	0	0	0	0	0	0	0	0	0	0.005	0.006	0.006
Total	1.7	2.1	2.5	1.6	2.0	2.4	1.4	1.8	1.3	1.0	1.3	1.6

Notes: LB—lower bound (analytical data <LOQ = 0); MB—middle bound (analytical data <LOQ = LOQ/2); UB—upper bound (analytical data <LOQ = LOQ).

**Table 6:** Estimation of average exposure of OTA in adult consumers (ng/kg bw/day).

Food group	Population Group											
	Men 18–59 years			Women 18–59 years			Men 60 and more years			Women 60 and more years		
	LB	MB	UB	LB	MB	UB	LB	MB	UB	LB	MB	UB
Cocoa	0.002	0.003	0.004	0.002	0.003	0.004	0.002	0.003	0.003	0.002	0.002	0.003
Coffee	0.039	0.042	0.046	0.055	0.059	0.064	0.043	0.047	0.051	0.044	0.048	0.052
Confectionery	0.010	0.020	0.020	0.010	0.020	0.020	0.004	0.004	0.006	0.006	0.007	0.008
Dried fruit	0.001	0.002	0.002	0.002	0.002	0.003	0.001	0.001	0.002	0.002	0.003	0.004
Grains and grain-based products	0.260	0.420	0.590	0.240	0.360	0.480	0.270	0.430	0.600	0.240	0.370	0.490
Juice	0.007	0.008	0.008	0.014	0.016	0.017	0.003	0.003	0.004	0.006	0.006	0.007
Meat and meat products	0.030	0.110	0.210	0.020	0.100	0.180	0.020	0.090	0.160	0.020	0.080	0.150
Spices, seasoning and legumes	0.200	0.210	0.210	0.129	0.130	0.133	0.270	0.270	0.280	0.130	0.130	0.134
Tea	0.359	0.360	0.362	0.440	0.450	0.450	0.410	0.410	0.410	0.420	0.430	0.430
Beer	0.560	0.560	0.570	0.072	0.073	0.0734	0.52	0.53	0.53	0.056	0.056	0.057
Wine	0.013	0.015	0.016	0.021	0.024	0.026	0.011	0.011	0.012	0.008	0.008	0.009
Total	1.5	1.8	2.0	1.0	1.2	1.5	1.6	1.8	2.1	0.9	1.1	1.3

Notes: LB—lower bound (analytical data <LOQ = 0); MB—middle bound (analytical data <LOQ = LOQ/2); UB—upper bound (analytical data <LOQ = LOQ).

The highest OTA dietary exposure for “average consumer” to OTA in this study was estimated at 3.3 ng/kg bw/day (LB—lower bound) and 3.9 ng/kg bw/day (MB—middle bound) in children 4–6 years old. Principal sources of exposure for this population group included tea, grain-based products, confectionery, meat and meat products, and fruit juices (orange, grape, and apple). The dietary exposure for “average consumer” to OTA for the population group of children 7–10 years was estimated at 2.2 ng/kg bw/day (LB) and 2.6 ng/kg bw/day (MB). The OTA dietary exposure for “average consumer” for the population groups of boys and girls 11–14 years (altogether) was estimated at 2.1 ng/kg bw/day (LB) and 2.0 ng/kg bw/day (MB) respectively. Dietary exposure for “average consumer” to OTA for the population groups of men and women 15–17 years (altogether) was estimated at 1.8 ng/kg bw/day (LB) and 1.3 ng/kg bw/day (MB) respectively. The dietary exposure for “average consumer” to OTA for the population groups of men and women 18–59 years old (altogether) was estimated at 1.7 (LB) and 1.2 ng/kg bw/day (MB) respectively. The dietary exposure for “average consumer” to OTA for the population groups of men and women 60 and more years (altogether) was estimated at 1.8 ng/kg bw/day (LB) and 1.1 ng/kg bw/day (MB) respectively. Beer was found to be the main contributor of OTA dietary exposure in men 18–59 years old and correspond to 0.56 ng/kg bw/day (MB) about 31% of the total OTA intake. Grain-based products and tea were identified as the main contributors of OTA exposure in women 18–59 years old with an intake of 0.360 ng/kg bw/day and 0.45 ng/kg bw/day respectively. These two food items represented about 68% of the OTA intake in these women. The OTA dietary exposure from coffee and wine is higher in the population group of women aged 18–59 compared to the other population groups, but it can be considered only as minor contributors for the total OTA intake (0.059 and 0.024 ng/kg bw/day respectively).

Estimation of OTA intake of “high consumers” (ng/kg bw/day) are presented in Table 7, Table 8 and Table 9.

**Table 7:** Estimation of OTA intake of “high consumers” (95th percentile) in children group (ng/kg bw/day).

Food group	Population group					
	Children 4–6 years			Children 7–10 years		
	LB	MB	UB	LB	MB	UB
Cocoa	0.48	0.60	0.73	0.29	0.40	0.44
Coffee	0.27	0.30	0.31	0.29	0.30	0.34
Confectionery	5.30	6.50	7.70	3.70	4.60	5.50
Dried fruit	0.70	1.30	2.10	0.40	0.70	1.00
Grains and grain-based products	14.70	19.80	24.60	9.10	12.90	16.40
Juice	1.10	1.20	1.30	0.86	0.94	1.00
Meat and meat products	0.70	3.10	5.50	0.90	3.30	5.90
Spices, seasoning and legumes	11.50	11.60	11.80	17.20	17.30	17.50
Tea	12.00	12.00	12.13	8.20	8.30	8.40
Beer	0	0	0	0	0	0
Wine	0	0	0	0	0	0

Notes: LB—lower bound (analytical data <LOQ = 0); MB—middle bound (analytical data <LOQ = LOQ/2); UB—upper bound (analytical data <LOQ = LOQ).

**Table 8:** Estimation of OTA intake of “high consumers” (95th percentile) in adolescents group (ng/kg bw/day).

Food group	Population group											
	Boys 11–14 years			Girls 11–14 years			Men 15–17 years			Women 15–17 years		
	LB	MB	UB	LB	MB	UB	LB	MB	UB	LB	MB	UB
Cocoa	0.130	0.200	0.220	0.540	0.600	0.740	0.240	0.280	0.330	0.070	0.100	0.120
Coffee	0	0	0	0.070	0.070	0.070	0.130	0.140	0.150	0.170	0.180	0.190
Confectionery	3.200	3.80	4.40	2.80	3.30	3.80	1.40	1.70	2.00	1.50	2.20	2.80
Dried fruit	0.050	0.100	0.070	0.040	0.100	0.100	0.007	0.009	0.010	0.240	0.500	0.740
Grains and grain-based products	8.20	11.10	13.50	7.40	10.10	12.80	6.90	9.10	11.40	4.00	5.80	7.60
Juice	0.480	0.520	0.560	0.370	0.400	0.430	0.470	0.510	0.550	0.300	0.330	0.350
Meat and meat products	0.360	1.700	3.100	0.250	1.100	2.000	0.300	1.400	2.600	0.300	1.400	2.500
Spices, seasoning and legumes	4.850	4.900	5.000	6.000	6.100	6.150	2.250	2.300	2.350	6.500	6.600	6.640
Tea	5.600	5.670	5.700	4.800	4.800	4.900	3.000	3.000	3.040	4.290	4.320	4.400
Beer	0	0	0	0	0	0	1.170	1.180	1.190	1.710	1.720	1.730
Wine	0	0	0	0	0	0	0.002	0.002	0.003	0.460	0.480	0.510

Notes: LB—lower bound (analytical data <LOQ = 0); MB—middle bound (analytical data <LOQ = LOQ/2); UB—upper bound (analytical data <LOQ = LOQ).

**Table 9:** Estimation OTA intake of “high consumers” (95th percentile) in adult group (ng/kg bw/day).

Food group	Population group											
	Men 18–59 years			Women 18–59 years			Men 60 and more years			Women 60 and more years		
	LB	MB	UB	LB	MB	UB	LB	MB	UB	LB	MB	UB
Cocoa	0.10	0.13	0.16	0.09	0.11	0.13	0.12	0.15	0.19	0.08	0.10	0.12
Coffee	0.21	0.23	0.24	0.26	0.28	0.30	0.20	0.21	0.23	0.23	0.25	0.26
Confectionery	2.40	2.90	3.40	2.00	2.40	2.80	0.55	0.68	0.81	0.75	0.95	1.16
Dried fruit	0.14	0.26	0.38	0.15	0.28	0.42	0.03	0.03	0.04	0.25	0.49	0.75
Grains and grain-based products	5.30	7.70	10.10	5.60	7.90	10.40	4.00	5.50	7.00	4.40	5.90	7.40
Juice	0.29	0.31	0.34	0.30	0.33	0.35	0.25	0.27	0.29	0.18	0.19	0.21
Meat and meat products	0.48	2.1	3.6	0.41	1.8	3.23	0.26	1.20	2.10	0.24	0.10	1.79
Spices, seasoning and legumes	10.90	10.90	11.00	22.50	22.60	22.90	12.70	12.70	12.90	7.20	7.20	7.30
Tea	2.66	2.68	2.70	3.60	3.70	3.70	3.20	3.20	3.30	3.00	3.00	3.10
Beer	2.58	2.60	2.62	1.38	1.39	1.40	2.58	2.59	2.61	0.85	0.85	0.86
Wine	0.75	0.82	0.89	0.80	0.88	0.95	0.42	0.46	0.49	0.60	0.65	0.70

Notes: LB—lower bound (analytical data <LOQ = 0); MB—middle bound (analytical data <LOQ = LOQ/2); UB—upper bound (analytical data <LOQ = LOQ).

The highest OTA dietary intake for “high consumers” in group of children aged 4–6 years old was estimated from grains and grain-based products between 14.7 ng/kg bw/day (LB) and 19.8 ng/kg bw/day (MB) followed by tea at 12.0 ng/kg bw/day (LB and MB) and spices at 11.50 ng/kg bw/day (LB) and 11.60 ng/kg bw/day (MB). The intake for this group via confectionery ranged from 5.3 ng/kg bw/day (lower bound) to 6.5 ng/kg bw/day (middle bound). In the other population groups the highest OTA intake for “high consumers” was due to grains (5.30 to 7.90 ng/kg bw/day) after spices for which the intake can reach 10.9 ng/kg bw/day and 22.60 ng/kg bw/day in men and women respectively. For men who are “high consumers” of beer, the OTA intake can reach 2.60 ng/kg bw/day (MB). “High consumers” of tea in women ingested 3.70 ng/kg bw/day.

It is important to say that for calculating the OTA dietary intake from black and fruit tea, a conservative approach was used, based on assumption that 100% of OTA were transferred from black and fruit tea to the infusion. The estimated dietary exposure to OTA from black and fruit tea have an uncertainty because of recent results on the transfer of OTA from black and fruit tea to the infusion. The transfer from black tea and fruit tea to the infusion was 34.8% ± 1.3% and 4.1% ± 0.2% respectively [12].

## 3. Discussion

### 3.1. Presence of OTA in Food

In total, amounts of OTA found in the foodstuffs are always below the maximum levels defined by the EU legislation [13]. Nevertheless, we have confirmed that OTA is present in a wide range of different food and, thus, under certain conditions, their consumption may lead to exceeding the tolerable daily intake (TDI).

#### 3.1.1. OTA in Cereal Products

OTA in cereal products (including rye bread, rye-wheat bread, multigrain bread and rolls, French bread, oat rolled grains, cereal porridge, fine bakery wares, breakfast cereals, sweet biscuits, sponge biscuits, cereal bars, and muesli) occurred in 28% of cereal products with an OTA amount ranging from ND to 2.14 ng/g (mean value 0.22 ng/g). The three highest concentrations in this category of food were identified in sponge biscuits (2.14 ng/g), in sweet biscuits (1.69 ng/g) and in muesli (1.44 ng/g). These data are more or less comparable with studies done in other Western/European countries.

For example, in the recent Spanish study, the OTA occurrence (%) and mean of positive samples [14] are the following: 2.8% and 0.728 ng/g in corn-based breakfast cereals; 25% and 0.293 ng/g in wheat-based breakfast cereals and 12.9% and 0.283 ng/g in loaf bread. In a Lebanese study [15], the food products exhibiting the highest concentration of OTA were cereal-based products, in particularly, “biscuits and croissants” (mean OTA concentration 2.844 ng/g). OTA was detected in 69% samples of breakfast cereals collected in France, with OTA the range of 0.2–8.8 ng/g. The highest concentration was determined in a sample containing dry fruit and bran [16]. Regarding contamination of bread, Duarte *et al.* [17] compiled the data on the occurrence of OTA in different types of bread worldwide. OTA detection frequency ranged from 65% to 100%. The mean values of positive samples of wheat bread ranged from 0.07 ng/g in Switzerland [18] to 13 ng/g in Morocco [19], although they are mostly below 0.50 ng/g. For comparison in our study, 21% of bread (French bread, multigrain bread, rye bread, and rye-wheat bread) were contaminated by OTA (mean value of 0.15 ng/g).

#### 3.1.2. OTA in Rice

The occurrence of OTA in rice in our study was 8% with an OTA level ranging from 0–3.76 ng/g (mean value 0.23 ng/g). These findings are compared to data reported from other countries.

In Morocco, 26% of samples of rice collected contained OTA (0.08–47 ng/g, average 3.5 ng/g). On this basis, the daily intake calculated as 0.32 ng/kg bw/day [20]. This value was two times higher than the estimated daily intake (0.17 ng/kg bw/day) calculated from organic and non-organic rice [21]. In the later study, 7.8% of non-organic rice contained 4.3–27.3 ng/g whereas 30% of organic rice contained 1–7.1 ng/g. In Vietnam, 35% of the samples of rice analyzed contained OTA (average value of 0.75 ng/g; maximum 2.75 ng/g). The contamination was higher in dry season compared to the rainy season [22]. In Nigeria, some rice samples were collected in field, in store, and in an open market. Altogether, 66.7% of the samples contained OTA in the range of 141.7 ± 25.4 ng/g with the highest amount of 341.3 ng/g. All samples from the store contained OTA (261.4 ± 22 ng/g); 70% of field samples contained 122 ± 30.8 ng/g whereas only 20% of the samples from market contained 37.6 ± 37.6 ng/g [23]. In China, 4.9% of rice contained at average of 0.85 ng/g of OTA with a maximum of 3.2 ng/g [24]. In a Lebanese study [15] the food products presenting high concentrations of OTA were “rice and rice based products” (mean OTA amount 0.680 ng/g).

#### 3.1.3. OTA in Cocoa and Chocolate-based Products

As for cocoa and chocolate-based products 33% were contaminated with OTA (with the mean value of 0.94 ng/g in cocoa powder, 0.29 ng/g in bitter chocolate, 0.29 ng/g in chocolate coated confectionery, 0.17 ng/g in milk chocolate and 0.13 ng/g in filled chocolate, and the highest contamination (4.1 ng/g) in cocoa powder.

These data are in the line with the Italian study, in which 300 samples of cocoa and chocolate-based products were randomly collected in South, Central, and Northern Italy [25]. One hundred and seventy nine, out of the 300 samples analyzed, were positive for OTA, representing 60% of the purchased products. All 40 cocoa samples contained OTA, while grouping all the chocolate products, the positive samples accounted for 53%. The mean OTA concentration for cocoa, dark chocolate bar, milk chocolate bar, chocolate candies, and Easter egg was 0.55 ± 0.51 ng/g, 0.20 ± 0.19 ng/g, 0.15 ± 0.07 ng/g, 0.15 ± 0.18 ng/g, and 0.20 ± 0.12 ng/g respectively [25]. In a small survey, 50% of cocoa powder were shown to contain OTA with values ranging from 0.22–0.77 ng/g and with the mean of 0.43 ng/g. Only the cocoa beans coming from conventional farms contained OTA [26]. The recent study by Copetti *et al.* [27] showed that 98% of purchased chocolate contained OTA. On average, the highest amounts of OTA were found in powdered, dark, and bitter chocolate at 0.39; 0.34 and 0.31 ng/g respectively. A survey in Canada showed that the incidences of OTA above the LOQ in natural cocoa were 100% (mean 1.17 ng/g), 95% for alkalized cocoa (mean 1.06 ng/g), 100% for baking chocolate (mean 0.49 ng/g), 100% for dark chocolate (mean 0.39 ng/g), 70% for milk chocolate (mean 0.19 ng/g), and 100% for cocoa liquor (mean 0.43 ng/g), but no contamination was found in cocoa butter [28]. In 2004, Serra Bonvehí [29] summarized the data on the occurrence of OTA in 170 samples of cocoa products of different geographical origins. The highest levels of OTA were detected in roasted cocoa shell and cocoa cake (0.1–23.1 ng/g) and only at minor levels in the other cocoa products. Twenty-six cocoa and chocolate samples were free from detectable OTA (<0.10 ng/g). In roasted cocoa powder, 38.7% of the samples analyzed contained OTA at levels ranging from 0.1 to 2 ng/g, and 54.8% was contaminated at >2 ng/g (and 12 samples at >3 ng/g). OTA was detected in cocoa bean at levels from 0.1 to 3.5 ng/g, the mean concentration being 0.45 ng/g; only one sample exceeded 2 ng/g (4.7%).

#### 3.1.4. OTA in Coffee Products

In our study, 75% of coffee products (ground, roasted and instant) were contaminated with OTA (the mean 0.73 ng/g, highest value 4.91 ng/g in instant coffee).

In Catalonia, 49% of coffee samples were contaminated with OTA with levels ranging from 1.21–4.21 and a mean 2.17 ± 0.79 ng/g [30]. In another study, 69% of coffee samples were contaminated to a different extent, depending of the type of coffee: 2.7 ng/g were found in green coffee, 0.24 ng/g in roasted coffee and 0.43 in soluble coffee [31]. In Italy, 50% of coffee samples contained OTA with values ranging 0.22–0.77 ng/g, with the mean of 0.43 ng/g [26]. In France, in all samples of ground coffee, traces of OTA were found. 27% of the samples contained OTA with values ranging from the limit of detection (LOD) LOD–0.5 ng/g; 33% from 0.5–1 ng/g; 37% 1–3 ng/g. One sample exceeded the EU regulatory limit and reached 11.9 ng/g [32]. In Chile, the amount of OTA found in roasted coffee ranged from 0.30–0.84 ng/g and between 0.28–5.58 ng/g in instant coffee [33]. Vecchio *et al.* [34] found OTA in 96% of instant coffee with values ranging from 0.32–6.40 ng/g. Casal *et al.* [35] found OTA in all soluble coffees ranging from LOD–11.8 ng/g with the mean of 2.49 ng/g. In a Lebanese study [16], the food products with the highest concentrations of OTA were described as “caffeinated beverages” (mean OTA concentration 0.508 ng/g).

A new method for the detection of OTA and other mycotoxins in coffee beverages by triple quadrupole and ion trap liquid chromatography tandem mass spectrometry was published [36]. LOD and LOQ were 0.24 to 0.42 ng/g, respectively. The recovery value was 94 ± 8%. The developed method was demonstrated in six real samples of roasted and instant coffee, caffeinated and decaffeinated coffee, and coffee with sugar added. The analyses indicate the presence of OTA in decaffeinated coffee (1.84 ng/g) and in instant coffee with sugar and milk added (4.93 ng/g) [36].

#### 3.1.5. OTA in Wine

In total of 24 samples of red and white wine were analyzed in our study. One third was contaminated with OTA; its levels ranged from 0.069 to 0.695, the mean was 0.069 ng/g (red wine); 0.020 (white wine).

Bellver Soto *et al.* [37] have recently published a review on the presence of OTA in red, rose, and white wine from Southern Europe. In red wine, OTA values of incidence ranged from 53% to 100% (range of OTA 0.02–7.5 ng/mL), in rose wine from 33% to 92% (range of OTA 0.4–4.07 ng/mL), and in white wine from 19% to 100% (range of OTA 0.17–27.8 ng/mL). In 2005, 267 wine samples (19 desert, 186 red, 11 rose, 51 white wine) from Italy and Hungary were analyzed. None of the Hungarian wines contained detectable amounts of OTA whereas 84% of Italian wines contained OTA with levels ranging from 0.01–4 ng/mL. The respective occurrence and mean values were 63% of desert wines (0.01–1.64 ng/mL), 56% rose (0.01–1.04 ng/mL), 19% white (0.01–0.21 ng/mL) [38]. A Spanish survey on the occurrence of OTA in 240 grape-based beverages was carried out. Red and white wines from four different Spanish Designations of Origin (*n* = 160), musts (*n* = 20), grape juices (*n* = 10), ordinary wines (*n* = 20), special wines (Malaga, muscatel, sherry, vermouth, *etc.*) (*n* = 20) and sparkling wines (*n* = 10) were assayed for OTA content. Forty-three (17.9%) of the samples tested contained detectable levels of OTA. The overall mean OTA concentration in red and white wines of Designations of Origin was 0.30 and 0.18 ng/mL respectively (ranges 0.05–3.19 and 0.05–1.13 ng/mL respectively). The percentage of wine samples with detectable amounts of OTA was higher for red (18.3%) than for white (10%) wines. OTA was also found in two of 10 red ordinary wines (0.68 and 4.24 ng/mL), whereas none of 10 white ordinary wines contained OTA. The mean OTA amount detected in sparkling wines was 0.44 ng/mL (range 0.14–0.71 ng/mL). Two of 20 must samples contained OTA at low levels (0.08 and 0.18 ng/mL), while none of 10 grape juice samples contained OTA. Highest amounts of OTA were found in special wines (45%), with a maximum of 15.25 ng/mL in a muscatel sample [39]. In another study, 95 Turkish wines (34 white, 10 rose, 51 red) were analyzed. Even though the mean values were similar in white and red wines (0.108 ng/mL *vs*. 0.110 ng/mL), the highest contamination was found in red wine (0.815 ng/mL), whereas the mean value in rose was 0.52 ng/mL [40]. The comparison of Moravian wines with foreign wines in a Czech study showed that OTA was present in 11% of Moravian wines (1.2–71.2 ng/mL); of 42% French wines; of 25% Italian wines; and in 100% Portuguese, Greek, Chilean, and South African wines, with the highest levels found in Greek wines [41]. All Greek sweet wines contained OTA ranging 0.05–2.30 ng/mL, the mean value 0.65 ng/mL. In the same study, 88.5% of dry wine contained OTA (mean value 0.19 ng/mL; range LOD–0.56 ng/mL); 76.2% of red wines (mean value 0.1 ng/mL, range LOD–0.71 ng/mL); 100% of rose (mean value 1.64 ng/mL, range 0.19–2.52 ng/mL) [42]. In a recent survey conducted for Dutch wines, only 4.8% of wine samples contained OTA (0.2–0.6 ng/mL) in 2008 whereas none of the wines sampled in 2009, 2010, 2011, 2012 were contaminated [43]. In a recent survey, it was demonstrated that other ochratoxins can be found simultaneously with OTA. Indeed 99% of the wines analyzed contained OTA ranging LOD–0.455 ng/mL and OTB (dechlorinated OTA) in the range of 0.0215–0.119 ng/mL. In 89.6% of samples OTC (ethylester OTA) was also detected (0–0.0315 ng/mL) [44].

#### 3.1.6. OTA in Beer

In our study, OTA was present in 91% of beer samples and in 100% of lager beer samples (the mean value 0.065 ng/g; the maximum value 0.26 ng/g).

Several years ago [45] 39% of the samples of beer were contaminated in the same range as the data obtained from the European Commission (SCOOP 3.2.7 2002) [46]. However, only one pale beer sample reached the value of 0.2438 ng/mL. Many studies on the occurrence of OTA in beer have been conducted [37,47,48,49,50,51]. In most cases, the mean OTA amounts were below 0.070 ng/mL, except for beer samples from Korea (0.25 ng/mL) [52], a sample of Scottish origin (0.201 ng/mL) [53], and Belgian beers (0.103 ng/mL) [48]. OTA occurrence ranged from 0 to 100%, but it often exceeded 50%. Bertuzzi *et al.* [54] analyzed 106 beer samples collected in 25 European countries. OTA occurred in 67.9% of samples and its concentrations ranged from <0.002 to 0.189 ng/mL, with a mean of 0.019 ng/mL. The latter value was lower than the mean value found in Spain (0.044 ng/mL) [55] and 0.0358 ng/mL [53]. Our data are similar to those found in the studies carried out in Eastern Central Europe, like in beers from Hungary where all samples contained small amounts of OTA except for one sample (0.25 ng/mL) [56].

#### 3.1.7. OTA in Spices

In our study 100% of fiery paprika powder, sweet paprika powder, nutmeg, and dried root licorice and 92% of dried chili pepper are contaminated by OTA (with mean value of 19.00 ng/g in fiery paprika powder, 16.00 ng/g in sweet paprika powder, 15.80 ng/g dried root licorice, 8.70 ng/g in nutmeg and 6.70 ng/g in dried chili pepper, and the highest contamination (91.80 ng/g) in fiery paprika powder. The occurrence of OTA in other spices (including black pepper, caraway seed, coriander seed, ginger dried root, and mixed spices e.g., curry powder) was 57% with an OTA level ranging from 0–12.70 ng/g (mean value 1.84 ng/g). The three highest concentrations in this category of food were in ginger dried root (12.70 ng/g), in mixed spices (e.g., curry powder) (9.40 ng/g) and in black pepper (2.82 ng/g). These data are more or less in the same range as studies done in other countries.

Paprika and chili were collected in Spain. 99% of paprika contained OTA in the range of <LOQ–281 ng/g; 100% chili samples contained OTA ranging 0.62–44.6 ng/g [57]. In chili samples collected in Malaysia in open market and supermarket, the occurrence of OTA was 81.25% with level ranging from 0.2–101.2 ng/g, average value 7.15 ± 17.30 ng/g; median value 3.92 ng/g. The samples collected in open market were more contaminated than the samples from supermarket [58]. Crushed chili samples are more often contaminated than powder or sauce [59]. Altogether a total of 170 samples (sauce, crushed chili, powdered chili) were collected in open market and supermarket. The amount of OTA ranged from 7.9 ± 1.9 ng/g in sauce collected in restaurant and 8.6 ± 1.2 ng/g with a maximum of 24.1 ng/g in sauce collected in open market. Concerning crushed chili the samples collected in open market contained OTA ranging 16.9 ± 2.1 ng/g with a max of 54.3 ng/g, and those collected in restaurant contained OTA ranging 19.8 ± 1.3 ng/g. In the chili powder the highest value of 64.5 ng/g was found in chili powder collected in the open market (OTA range 21.4 ± 1.9 ng/g). The contamination was similar in chili powder from restaurant (22.9 ± 2.8 ng/g; max 58.1 ng/g) [59]. In Sri Lanka, 41% of chili products are contaminated. 11% of chili pods contain OTA (<LOQ–5.3 ng/g); 50% of chili flakes contained <LOQ–15 ng/g, average amount 4.9 ng/g and; 48% of chili powder contained <LOQ–282 ng/g, average 16 ng/g [60]. Ozbey *et al.* [61] found also OTA in 75% of red chili flakes (0.46–53.04 ng/g, mean 16.44 ng/g); 54.5% powder chili (0.78–98.2 ng/g); 17.4% black pepper powder (0.87–3.48 ng/g, mean 1.82 ng/g). In another study, several spices and aromatic herbs were collected in Poland and 49% were contaminated by OTA [62]. All samples of black pepper grain, garden thyme, curry, and oregano contained OTA ranging 23.57 ± 2.61ng/g, 15.59 ± 2.84 ng/g, 19.01 ± 6.97 ng/g, 22.12 ± 1.77 ng/g, respectively. Only 80% of ground pepper, 85.7% of white pepper, and 62.5% of cayenne pepper were contaminated to 9.46 ± 0.8 ng/g, 29.41 ± 1.86 ng/g, 45.64 ng/g respectively. Cinnamon (72%), garlic (66.7%), marjoram (80%), and rosemary (50%) are less contaminated: 2.14 ± 0.7 ng/g; 0.11 ± 0.03 ng/g; 7.13 ± 0.17 ng/g; 5.07 ± 0.18 ng/g respectively. No OTA was found in nutmegs [62]. Pepper, chili, prickly ash, cinnamon, aniseed, fennel, curry powder, and cumin were collected in retail store in China. On a total of 480 samples, 9.6% contained OTA. The highest contamination was in chili (19.9%, mean 6.7 ng/g, max 30.73 ng/g). Ten percent of prickly contain an average of 3.17 ng/g (max 9.82 ng/g). Five percent of cinnamon contained OTA (mean 1.1 ng/g; max 2.02 ng/g. Curry (45.5%) and cumin (24.1%) contained also OTA (mean 2.44 ng/g, max 6.24) [63]. A total of 130 samples of spices coming from India, China, South America, USA, North Africa, Europe, and sub–Sahara Africa were collected in Italy. OTA was found in 60% of chili samples in the range of 2.16–16.35 ng/g, and in 13.3% of pepper in the range of 1.61–15.85 ng/g [64].

#### 3.1.8. OTA in Nuts

OTA was not detected in dried chestnuts, hazelnuts, or walnuts in our study. All results obtained were under the limit of quantification (<0.2 ng/g). No OTA was found in dried chestnut whereas 5/32 fresh nuts contained an average of 0.34 ± 0.19 ng/g with the highest value of 6.44 ng/g. The occurrence of OTA in chestnut flour was 68%, mean OTA amount 0.87 ± 2.00 ng/g and highest value of 9.99 ng/g [65]. Tiger–nuts can be contaminated with OTA in the range of 1.2–37 ng/g [66,67]. Peanuts are also frequently (85.7%) contaminated with OTA, the mean value of 3.7 ng/g, the highest amount of 4.9 ng/g [68]. By way of comparison, the OTA occurrence (%) and the mean of positive samples in a Spanish study [14] were the following: 41.7% and 0.241 ng/g in peanuts, and 2.9% and 0.228 ng/g in pistachios. In a Lebanese study [15], the food products with the lowest concentrations of OTA included nuts, seeds, olives, and dried dates (mean OTA concentration 0.078 ng/g).

#### 3.1.9. OTA in Meat and Meat Products

Only a few meat products (3.3%) which have been analyzed in the present study have been contaminated with OTA (mean value 0.12 ng/g). The highest concentration has been found in pork kidney (0.46 ng/g).

In the Canadian Total Diet Study from 2008–2009 [69], samples of fresh pork meat, cured pork meat, canned luncheon meat, poultry meat, lamb meat, sausages, eggs and cheese were analyzed by LC-MS/MS. OTA was found only in the samples of fresh pork meat (OTA concentrations 0.03 and 0.23 ng/g), cured pork meat (OTA concentrations 0.06 and 0.20 ng/g) and sausages (OTA levels 0.12 and 0.06 ng/g). In Serbia [70], 26.6% of pig liver contained OTA in the range of 0.22–14.5 ng/g. The data on the occurrence of OTA in pork kidneys were very similar (33.3%), with a maximum concentration of 52.5 ng/g. The majority of tissues samples from chicken did not contain measurable amounts of OTA. In Portugal, 25% of pig samples were contaminated in the range of <LOD–0.405 ng/g with a maximum of 0.578 ng/g [71]. In Pakistan, the contamination of chicken meat was higher compared to other countries. In broiler chicken, 41% of samples contained OTA with the mean values as follows: 1.39 ± 0.78 ng/g (wings); 0.28 ± 0.79 ng/g (chests); 1.12 ± 0.19 ng/g (legs); 2.21 ± 0.43 ng/g (liver). In layer chicken, 48% of samples contained OTA with mean values as follows: 1.45 ± 0.24 ng/g (wings); 0.81 ± 0.14 ng/g (chests); 1.59 ± 0.67 ng/g (legs); 2.41 ± 0.72 ng/g (liver) [72]. In Egypt, beef luncheon meat and beef burger collected in a supermarket were contaminated both by aflatoxin and OTA, with the mean values of 5.23 ng/g of OTA for beef luncheon meat and 4.55 ng/g for burger [73]. All of the dry cured hams analyzed contained OTA be it even in the deep section of hams, with values reaching 28.4 ± 0.12 ng/g. On the superficial portion, the concentration can be as high as 160.9 ± 0.48 ng/g [74]. Two studies conducted in Croatia analyzed meat products [75,76]. Sausages (*n* = 15), semi dry sausages (*n* = 25), and fermented dry meat products (*n* = 50) collected in supermarket were contaminated with OTA. The occurrence of OTA was 64.44% with the highest amount 7.83 ng/g in ham sausage, 2.37 ng/g in rabbit sausage, 2.03 ng/g in deer sausage, 3.07 ng/g in wild boar sausage, 1.37 ng/g in roe deer sausage and 2.71 ng/g in mixed sausage (wild boar, deer and pork). In semi dry sausage 84% contained OTA: 1.51–1.86 ng/g (grill sausage), <LOD–3.28 ng/g (Kranjska), 2.03–2.31 (Slavonian), 1.23–1.62 ng/g (Zagorska), <LOD–2.12 ng/g (homemade garlic). In dry meat 54% contained OTA: 2.03–2.31 (domestic Slavonian), <LOD–4.05 ng/g (home-made mixed pork & beef), 1.53–2.83 ng/g (homemade dry Zagorje), <LOD–7.83 ng/g (Salami) [75]. In another study, 105 hams, 208 dry fermented sausages, 62 bacon, and 35 cooked sausages collected in 2011–2014 were analyzed. Concerning ham, 20% of Istrian prosciutto contained OTA (mean value 0.78 ng/g, max 9.42 ng/g); 14.8% of Dalmatian prosciutto (the mean 0.33ng/g, max 3.16); 13.6% mean 0.16, max 1.29 ng/g; 33.3% other origins mean 1.82 ng/g, max 9.95 ng/g. In the dry fermented sausages, 5.7% Slavonski kulen contained OTA (mean 0.08 ng/g; max 1.52 ng/g); 7.1% of Istrian type mean 0.14 ng/g, max 2.64; 6.6% Slavonian type mean 0.11 max 2.35; 7.2% northern type mean 0.21 ng/g max 5.10 ng/g. 59% of Slavonian bacon contained OTA mean value 0.07 ng/g max 1.23 ng/g. Three percent of cooked sausage (liver) contained OTA (the mean 1.1 ng/g, max 3.13 ng/g) [76].

### 3.2. Dietary Exposure Assessment

The exposure to OTA is a function of the concentration of OTA in foodstuffs, as well as the amount of these foodstuffs that are consumed in different populations. Using the ranges of OTA contamination of food, OTA exposure has been estimated from the known intake amounts of the given commodities.

The herein estimated sum of average dietary intakes (3.9 ng/kg bw/day for children 4–6 year old; 2.6 ng /kg bw/day for children 7–10 years old; 2.1 ng/kg bw children 11–14 years old; 1.8 ng/kg bw/day for males above 15 years old; about 1.2 ng/kg bw/day for females above 15 years old) were lower, albeit close to the tolerable daily intake (TDI) as recommended by the European Commission’s SCF at 5 ng/kg bw/day in 1998 [77] and far below the one proposed by the Joint FAO/WHO Experts Committee on Food Additives [78] of 14.28 ng/kg bw/day. However, it exceeds virtually safe dose of 1.5 ng/kg bw/day calculated by Kuiper–Goodman and Scott [79] and is very close to the TDI of 4 ng/kg bw/day as recently calculated by Kuiper-Goodman *et al.* [9].

For children aged 4–6 and 7–10, the major contributors of OTA included tea (49% and 43%), grain-based products (28% and 32%) followed by confectionery (7% and 8%), spices (5% and 8%), meat products (5% and 6%) and juices (3% and 3%). In adolescents, the major contributors of OTA were tea (41%), grain-based products (35%) followed by spices (8%), meat products (8%), confectionery (4%) and juice (1.7%).

In the group of the adult men, the major contributors to OTA were beer (30%), grain-based products (24%) and tea (21%) followed by spices (13%), meat products (6%) and coffee (2.5%). Wine is only a minor contributor (0.7%). In adult women, the major contributors of OTA consisted in tea (38%), grains and grain-based products (32%), spices (11%), meat products (8%), beer (5.6%), coffee (4.7%) and wine (1.4%).

The fact that the majority of the calculated intakes are below the tolerable doses should not be neglected. The calculation is based on an average value which means that some individuals may exceed this value, and so be at a risk. Estimations for the 97.5th percentiles indicated that the ingestion of some foodstuffs leads to an OTA intake exceeding the recommendation.

In children, some favourite food may account for a high amount of OTA: with grain based products 19 ng/kg bw/day (children aged 4–6 years) and 12.9 ng/kg bw/day (aged 7–10 years); tea 12 ng/kg bw/day (children 4–6 year old) and 8.3 ng/kg bw/day (7–10 years old); spices 11.6 ng/kg/day (children 4–6 year old) and 17.3 ng/kg bw/day (7–10 years old). In adolescent and adults, high consumers of grain based product can ingest amounts as high as 11.1 ng/kg bw/day (male adolescent). In female (18–60 years old), spices can account for 22.6 ng/kg bw/day.

By way of comparison, the most recent international exposure assessments were performed respectively by the SCF [77] and the JECFA [78]. The SCF estimated that the mean dietary intake ranged from 0.7 to 4.6 ng/kg bw/day. By combining the average contamination levels with the 95th percentile of food consumption, the JECFA estimated a dietary exposure at approximately 90 ng/kg bw/week corresponding to about 13 ng/kg bw/day. The exposure seems to be predominantly associated with the consumption of contaminated plant-derived products and only to a minor extent to food of animal origin [80]. However, it cannot be excluded that regular consumption of certain regional specialties, of both plant origin, e.g., traditional Turkish food (containing nuts, dried fruit, and grape juice as ingredients) [81] and animal origin, e.g., Swedish blood pudding containing substantial amounts of pig blood [82] may contribute considerably to the level of exposure, especially in children, in which the relatively lower body weight as compared to adults, results in a higher exposure per kg bw.

A similar result was obtained in a one-month duplicate diet study which was performed in the UK. Analysis of the food indicated OTA levels contributing to a mean OTA intake at 65 ng/day and 0.9 ng/kg bw/day, respectively, in the range 0.26–3.54 ng/kg b.w/day over the 30 days [83].

Exposure levels for average adult consumers (2–3 ng/kg bw/day) and high adult consumers (6–8 ng OTA/kg bw/day) were established using the recent SCOOP data [46] from all the EU Member states and the draft EFSA concise database on food consumption in three representative EU member states (France, Italy, and Sweden). These estimates are considered to be conservative for the adult EU population as broad food categories were used in the assessment of consumption [8].

The results from the first French Total Diet Study show that the estimated average intake of OTA in the French population was 2.2 ng/kg bw/day for adults and 4.1 ng/kg bw/day for children. The 95th percentile exposure is 3.6 ng/kg bw/day in adults and 7.8 ng/kg bw/day in children. The food groups contributing most (70%) to this exposure of both population groups are cereals and cereal products (bread, rusk, breakfast cereals, pasta, rice and semolina, other cereals, Viennese bread, biscuits and cakes); other food groups, mainly grape-based products (dried raisins, raw grapes, grape juice and wine), coffee, nuts, and oilseeds contribute for less than 5% to the total exposure [84]. These data confirmed the estimation made in 1999 that showed an average exposure of the adult of 1.3 ng/kg bw/day, and 6.9 ng/kg bw/day for the 95th percentile of children [10].

The results from the second French Total Diet Study [85] show that the exposure of the French population in lower bound (LB) was generally concordant with the data from Norwegian, Swedish, and Spanish consumers [14,82], based on the combination of consumption data from food frequency questionnaires and contamination data or from the literature, or from analysis of pooled samples. The estimation of exposure from a 2005 Dutch duplicate diet survey (1.2 ng/kg bw/day) was also in the range of our LB-UB results [86]. The upper bound (UB) exposure (mean and 95th percentile) was close or lower than a North American assessment of usual exposure based on a probabilistic approach [9]. Another study concluded that these exposure levels were not associated with a significant cancer risk, based on the animal lowest observed adverse effect limit and the tumorigenic dose associated with a 5% increase in tumor incidence above background [87].

In an Italian study [25], “teenagers’ male group” resulted as the highest consumers for cocoa powder (2.9 g per day), while the highest OTA weekly intake were reported for the younger age groups (0.10 and 0.38 ng/kg bw/week or 0.014 and 0.054 ng/kg bw/day, for infant and children respectively). With respect to chocolate products, the highest consumption and the highest intake are reported for children (7.3 g and 0.57 ng/kg bw/week or 1.04 g and 0.081 ng/kg bw/day, respectively). As far as single chocolate products are concerned, the highest weekly intake is referred to the consumption of the Easter chocolate eggs by the infant/children age group (0.10 ng/kg bw/week or 0.014 ng/kg bw/day).

In another study from China, the data of the dietary intake of OTA on the adult inhabitants in the city of Shanghai were presented. The results from the point evaluation indicated that the mean value of daily intake of OTA was 1.147 ng/kg bw/day and the high percentile (97.5th) value of daily intake of OTA was 8.566 ng/kg bw/day. In the different groups of food, OTA in cereals and derived products was the largest contribution to the potential health risk. The mean daily intake and 97.5th percentile daily intake were 1.093 and 7.962 ng/kg bw/day, respectively, indicating that more than 90% of the risk was due to the contamination of OTA in cereals and derived products, which were the major food for the inhabitants in Shanghai. The mean daily intake and 97.5th percentiles daily intake of OTA in beans based-products were 0.029 ng/kg bw/day and 0.354 ng/kg bw/day, respectively. In dried fruits products mean daily intake was 0.019 ng/kg bw/day and 95th percentiles intake was 0.210 ng/kg bw/day. Although OTA was frequently found in grapes and derived products, relatively low intakes were observed (mean daily intake 0.005 ng/kg bw/day and 97.5th percentile 0.040 ng/kg bw/day) [88].

In Portugal, the total population dietary intake was calculated at 0.81 ng/kg bw/day, with wheat and white (wheat) flour being the most important source of the studied food groups since it accounted for 0.69 ng/kg bw/day (~85%). The remaining contributions studied included (in ng/kg bw/day) coffee (0.09), wine (0.02), and beer (0.01). The mean body weight was set at 65 kg. To increase reliability, all the consumption data were retrieved from the official Institute of Statistics of Portugal, and all of them corresponded to the period/year of the sampling and analysis of each commodity. The estimated sum of dietary intake was calculated at 3.979 ng/kg bw/day [17].

In Egypt, the assessment of human exposure to OTA was achieved by analyzing some foodstuffs, food consumption from a series of individuals (25 volunteers) using diet duplicate method, and urinary OTA concentration. The average daily intake ranged from 1.07–8.43 ng/kg bw/day, with a mean value of 4.49 ± 1.95 ng/kg bw/day [89].

## 4. Materials and Methods

### 4.1. Sampling of Foods

Foods of both plant and animal origin (86 kinds) were collected in five sampling terms in 12 regions of the Czech Republic in years 2011–2013. Eighty percent of foods were purchased in urban areas while 20% in the rural ones in line with the data of consumer behavior in the Czech Republic. In total, 1032 food samples were gathered.

### 4.2. OTA Chemical Analysis

Validated and accredited ultra-trace HPLC-FLD method was employed for OTA determination [12,13,90]. The food samples were extracted by solvent according to the food matrices and cleaned by means of immunoaffinity chromatography (OCHRAPREP^®^ columns, R-Biopharm, Darmstadt, Germany) as described by Skarkova *et al.* [90] and Malir *et al.* [12,13]. The limit of quantification of the method (LOQ) varied between 0.01–0.2 μg/kg, recoveries of 75%–90% and relative standard deviation of repeatability (RSD_r_) 1.4%–5.7% to the type of sample matrix.

The laboratory of National Reference Center for Microfungi and Mycotoxins in Food Chains is accredited accordance to CSN EN ISO/IEC 17025:2005 [91]. The reference materials P64/OW 806 (OTA naturally contaminated wheat 7.7 μg/kg) and P64/OW 815 (OTA naturally contaminated wheat 4.9 μg/kg) were used for validation of the method. Samples of various food matrices were spiked with OTA and analyzed as test materials to confirm the quality of laboratory results. Repeatability of the method was examined by analyzing of the spiked food samples. In accordance with the CSN ISO 3534–1, results of repeatability were obtained with the same method on identical testing samples in the same laboratory by the same operator using the same equipment within short intervals of time [92].

The quality of laboratory results was confirmed by successful participation in international inter-laboratory proficiency testing (FAPAS, FERA, Sand Hutton, UK) in 2012 (Table 10).

**Table 10:** Results and z-score of FAPAS proficiency testing.

FAPAS^®^ Report No	Name	Assigned Value (μg/kg)	Results (μg/kg)	Recovery (%)	z-score ^a^	Laboratory Number
17106	OTA in red wine	0.91	0.95	82	0.2	32
17107	OTA in wheat flour	3.67	2.98	85	−0.9	32
17108	OTA in coffee	6.14	5.06	83	−0.8	18
17110	OTA in cocoa	10.3	11.5	83	0.5	9

Note: ^a^ The interpretation for the z-score is the following: z-score ≤ 2 gratifying results; 2 < z-score ≤ 3 questionable results; z-score ≥ 3 degratifying results.

A difficult step in the dietary exposure assessment is how to handle data on concentrations reported below the limit of quantification (LOQ) but over the limit of detection (LOD). These data are known as non-detected and the resulting distribution of the occurrence values is left-censored. Left-censored data management was done by substitution [93,94,95]. Three scenarios were considered: (i) the lower bound (LB) approach by replacing the results below LOQ by zero; (ii) the middle bound (MB) approach by replacing the results below LOQ by LOQ divided by 2; (iii) the upper bound (UB) approach by replacing the results below LOQ by LOQ.

GEMS-Food Euro [93] prefers, for left-censored, data the middle bound (MB) approach, EFSA [94] prefers the combination of the lower bound (LB) and upper bound (UB) approaches and Sand *et al.* [95], prefer the combination of the lower bound (LB) and middle bound (MB) approaches.

### 4.3. Food Consumption Data

#### 4.3.1. SISP04

Data from the national wide dietary survey (SISP04) were used for calculations of the dietary exposure [96]. The survey was conducted between November 2003 and November 2004 by the means of a repeated 24 h recall on an age and gender representative sample of the Czech population. Respondents were asked to report all food and beverages consumed over a 24 h period (from midnight to midnight). Two interviews were completed with each subject in non-consecutive days because the consumption data from at least two days should be available to perform a long-term exposure assessment. Participants were selected by multistage sampling combining random and quota selection based on statistics from the National Population and Housing Census of 2001. The overall response rate was 54%. Data collection was proportionally distributed during the whole year to limit the influence of seasonality in the consumption and covered all days of the week. The amounts consumed were estimated using either photos of portions for the most frequently consumed meals, household measures, or by using measuring guides.

The overview of population groups for which food consumption values were calculated is presented in Table 11.

**Table 11:** Numbering and body weight of 10 population groups aged 4–90 years, both sexes in Czech Republic.

No.	Population Group	Body Weight (kg)
1	Children 4–6 years	21.4
2	Children 7–10 years	32.2
3	Boys 11–14 years	46.9
4	Girls 11–14 years	45.3
5	Men 15–17 years	65.6
6	Women 15–17 years	55.6
7	Men 18–59 years	82.5
8	Women 18–59 years	67.7
9	Men 60 and more years	82.9
10	Women 60 and more years	75.2

The food consumption data are available on web [96].

The Czech food consumption data (SISP04) were used as reported to the EFSA Comprehensive European Food Consumption Database. The Comprehensive Food Consumption Database is a source of information on food consumption across the EU contains detailed data for a number of EU countries [97].

#### 4.3.2. FoodEx2

EFSA has developed a preliminary standardized food classification and description system called FoodEx2 (version 2 of the EFSA Food classification and description system for exposure assessment). The system consists of descriptions of a large number of individual food items aggregated into food groups and broader food categories in a hierarchical parent-child relationship. FoodEx2 was used e.g., to codify all foodstuffs and beverages present in “EFSA Comprehensive European Food Consumption Database” [98]. FoodEx2 was used for food description, classification, and aggregation in the study.

Aggregation of individual food items into food groups in our study are presented in Table 12.

**Table 12:** Aggregation of individual food items into food groups.

No.	Food Group
1	Cocoa ^a^
2	Coffee ^b^
3	Confectionery ^c^
4	Dried fruit ^d^
5	Grains and grain-based products ^e^
6	Juice ^f^
7	Meat and meat products ^g^
8	Spices, seasoning and legumes ^h^
9	Tea ^i^
10	Beer ^j^
11	Wine ^k^

Notes: ^a^ Cocoa powder, cocoa (beverage preparation powder); ^b^ coffee ground (roasted), instant coffee; ^c^ chocolate (coated confectionery), sweet confectionery, bitter chocolate, milk chocolate, filled chocolate; ^d^ dried wine fruits (raisins), dried figs; ^e^ rye-wheat bread, refined flour, French bread, fine bakery wares, multigrain bread and rolls, crackers, breakfast cereals, biscuits (sweet, plain), sponge biscuits (plain), cereal bars, biscuits (chocolate), gingerbread, muesli and similar products, oat rolled grains, pasta and similar products, rice grains; ^f^ fruit juices; ^g^ swine fresh meat, swine kidney, bird offal, chicken fresh meat; ^h^ paprika powder (sweet), paprika powder (fiery), chili pepper (dried), caraway, black pepper, nutmeg, mixed herbs and spices, mustard, beans; ^i^ tea leaves and stalks (fermented), green tea leaves and stalks (dry), herbs and vegetables for infusions (fruit tea), herbs and vegetables for infusions (herbal tea); ^j^ beer (regular), beer (regular lager); ^k^ wine (white), wine (red).

### 4.4. Dietary Exposure Assessment

The analytical data served as a basis for an assessment of dietary exposure of OTA for 10 population groups of age 4–90 years, both sexes.

The dietary exposure assessment has been based on a deterministic approach (point estimate—an analysis by simple distributions). The chronic dietary exposure of OTA in ng/kg bw/day or week was calculated by multiplying of the arithmetic mean of analytical results (using lower bound (LB), middle bound (MB) and upper bound (UB) approach) with the average value of food intake in population groups of interest

*DE=C_f_ × V_f i_*

where *DE* is the dietary exposure (ng/kg bw/day); *C_f_* is the arithmetic mean (LB, MB, UB) of analytical data of OTA in foodstuffs (ng/kg); *V_f i_* is the average and/or 95th percentile values of food intake in the population group of interest (kg/kg bw/day) [96].

## 5. Conclusions

The present work has assessed the dietary exposure to OTA in 10 age groups in the Czech population groups to OTA by determining the levels of contamination of certain food collected in Czech Republic, and by considering data of consumption for these populations groups. This study has shown that for some groups of the population (“young children” notably) the OTA intake is not insignificant as the average OTA amount exceeds the virtually safe dose of 1.5 ng/kg bw/day established by Kuiper-Goodman and Scott [79] and very close to the TDI of 4 ng/kg bw/day as recently calculated by Kuiper-Goodman *et al.* [9] and that shall protect from the cancer risk. Grain-based products for all populations represent the main contributor of dietary exposure and constitute a major risk for high consumers. Particular attention must be also devoted to spices and tea. In all the groups, the high consumers ingested amounts of OTA above the recommended intake.

A more complete dietary exposure assessment could be arrived at if other foodstuffs were included which were previously shown to be contaminated by high levels of OTA such as dried licorice root, dried ginger root, or coriander seed. These were, however, not included in OTA dietary exposure of “high consumers” because the consumption data are lacking.

It must be added that the use of probabilistic modelling techniques within the field of dietary exposure assessment is a subject of increasing interest. The next step might be probabilistic risk assessment of the dietary exposure to OTA in the Czech populations group by employing “MCRA (Monte Carlo Risk Assessment)” software.

## Abbreviations

OTA, ochratoxin A; LB, lower bound; MB, middle bound; UB, upper bound; DI, daily intake; LOQ, limit of quantification; LOD, limit of detection; HPLC-FLD, high-performance liquid chromatography with fluorescence detection; RSDr, relative standard deviation of repeatability; FAPAS, International food analysis proficiency testing services; FERA, The food and environment research agency; UK, United Kingdom; EFSA, European food safety authority; MCRA, Monte Carlo Risk Assessment.
